# Supplementary material for: Human Albumin Infusion in Critically Ill and Perioperative Patients: Narrative Rapid Review of Meta-Analyses from the Last Five Years
Source: J Clin Med. 2023 Sep 12;12(18):5919. doi: 10.3390/jcm12185919 (PMC10532105; doi:10.3390/jcm12185919)
Supplement: Supplementary file 1 [file jcm-12-05919-s001.zip › jcm-2586358-supplementary.pdf]

Supplementary Materials

Table S1. Excluded publications and reason for exclusion

1	Ashour AA, Atta MA, Sadek KW, Obaid KR, Ashour MA, Ashour A, Danjuma MI, Doi SA, and ElZouki AN. „Albumin administration in patients with decompensated liver cirrhosis: a meta-analytic update.“ European journal of gastroenterology & hepatology 33, Nr. 4 (April 2021): 479–86. https://doi.org/10.1097/MEG.0000000000001932.	Liver
2	Bai Z, Wang L, Cheng G, Lin H, and Qi X. „Use of human serum albumin infusion for the prevention and treatment of hyponatremia in liver cirrhosis: A systematic review and meta-analysis“. Hepatology International 16 (2022): S321–22. https://doi.org/10.1007/s12072-022-10337-4.	Liver
3	Bai Z, Wang L, Lin H, Tacke F, Cheng G, and Qi X. „Use of Human Albumin Administration for the Prevention and Treatment of Hyponatremia in Patients with Liver Cirrhosis: A Systematic Review and Meta-Analysis“. Journal of Clinical Medicine 11, Nr. 19 (2022). https://doi.org/10.3390/jcm11195928.	Liver
4	Bai Z, Wang L, Wang R, Zou M, Méndez-Sánchez N, Romeiro FG, Cheng G, and Qi X. „Use of human albumin infusion in cirrhotic patients: a systematic review and meta-analysis of randomized controlled trials.“ Hepatology international 16, Nr. 6 (Dezember 2022): 1468–83. https://doi.org/10.1007/s12072-022-10374-z.	Liver
5	Barsaga M L.A, Payawal D A, and Te M T. „Meta-analysis: Albumin infusion in patients undergoing large volume paracentesis“. Hepatology International 13 (2019): S227. https://doi.org/10.1007/s12072-019-09936-5.	Liver
6	Faustino J, Gorospe J D, Cua I H, and Cataluña J G. „Albumin infusion plus standard therapy in increasing survival among patients with acute-on-chronic liver failure secondary to infection: a systematic review and meta-analysis“. Hepatology International 17 (2023): S95–96. https://doi.org/10.1007/s12072-023-10501-4.	Liver
7	Hamza M, Jasti J R, Arshad N, Perez A M, Iltaf Satti, D, Aiman W, et al. „Progress in Drug Therapy for Hepatorenal Syndrome: A Systematic Review of Clinical Studies in the Last 3 Years“. American Journal of Gastroenterology 117, Nr. 10 (2022): S901. https://doi.org/10.14309/01.ajg.0000861628.70876.99.	Liver
8	Is B, Bombassaro IZ, Tovo CV, de Mattos ÂZ, Ahlert M, Chiesa T, and de Mattos AA. „Albumin in the management of hepatic encephalopathy: A systematic review and meta-analysis.“ Annals of hepatology 26 (Dezember 2021): 100541. https://doi.org/10.1016/j.aohep.2021.100541.	Liver
9	Kulkarni A V, Kumar P, Singh S, Sharma M, Talukdar R, Murthy V H.V, Singh V, Reddy N D, and Rao N P. „Prevention of paracentesis-induced circulatory dysfunction—A systematic review and network meta-analysis“. GastroHep 2, Nr. 3 (2020): 92–101. https://doi.org/10.1002/ygh2.395.	Liver
10	Kulkarni A V, Padaki Rao, and N. „Midodrine or albumin in paracentesis-induced circulatory dysfunction: Author’s reply“. GastroHep 2, Nr. 3 (2020): 138–39. https://doi.org/10.1002/ygh2.402.	Liver
11	Larrazabal R B, Chiu H H.C, and Yasay E B. „The effect of long-term human albumin administration on the mortality of adult patients with decompensated liver cirrhosis: A meta-analysis and systematic review“. Journal of	Liver

	Gastroenterology and Hepatology 36, Nr. SUPPL 2 (2021): 74. https://doi.org/10.1111/jgh.15607.	
12	Larrazabal R. „The effect of long-term human albumin administration on the mortality of adult patients with decompensated liver cirrhosis: A meta-analysis and systematic review". Hepatology International 14 (2020): S394. https://doi.org/10.1007/s12072-020-10030-4.	Liver
13	Leache L, Gutiérrez-Valencia M, Saiz L C, Uriz J, Bolado F, García-Erce J A, Cantarelli L, and Erviti J. „Meta-analysis: Efficacy and safety of albumin in the prevention and treatment of complications in patients with cirrhosis". Alimentary Pharmacology and Therapeutics 57, Nr. 6 (2023): 620–34. https://doi.org/10.1111/apt.17344.	Liver
14	Leão GS, John Neto G, Jotz RF, Mattos AA, and Mattos ÂZ. „Albumin for cirrhotic patients with extraperitoneal infections: A meta-analysis." Journal of gastroenterology and hepatology 34, Nr. 12 (Dezember 2019): 2071–76. https://doi.org/10.1111/jgh.14791.	Liver
15	Malik A and Amjad W. „EFFICACY AND SAFETY OF TERLIPRESSIN AND ALBUMIN VS NORADRENALINE AND ALBUMIN IN ADULT PATIENTS WITH HEPATORENAL SYNDROME: A SYSTEMATIC REVIEW AND META-ANALYSIS". Hepatology 76 (2022): S1139–40. https://doi.org/10.1002/hep.32697.	Liver
16	Mohamed M M.G, Rauf A, Adam A, Kheiri B, Lacasse A, and El-Halawany H. „Terlipressin effect on hepatorenal syndrome: Updated meta-analysis of randomized controlled trials". JGH Open 5, Nr. 8 (2021): 896–901. https://doi.org/10.1002/jgh3.12600.	Liver
17	Nanda A, Reddy R, Safraz H, Salameh H, and Singal A K. „Pharmacological Therapies for Hepatorenal Syndrome". Journal of Clinical Gastroenterology 52, Nr. 4 (2018): 360–67. https://doi.org/10.1097/MCG.0000000000000913.	Liver
18	Sandi BB, Leão GS, de Mattos AA, and de Mattos ÂZ. „Long-term albumin administration in patients with cirrhosis and ascites: A meta-analysis of randomized controlled trials." Journal of gastroenterology and hepatology 36, Nr. 3 (März 2021): 609–17. https://doi.org/10.1111/jgh.15253.	Liver
19	Shrestha D B, Budhathoki P, Sedhai Y R, Baniya R, Awal S, Yadav J, Awal L, Davis B, Kashiouris M G, and Cable C A. „Safety and efficacy of human serum albumin treatment in patients with cirrhotic ascites undergoing paracentesis: A systematic review and meta-analysis: Meta-analysis of albumin infusions in ascites due to cirrhosis". Annals of Hepatology 26 (2021). https://doi.org/10.1016/j.aohep.2021.100547.	Liver
20	Shrestha D B, Budhathoki P, Sedhai Y, Baniya R, Awal S, Yadav J, and Awal L. „Efficacy and safety profile of human serum albumin in patients with ascites due to cirrhosis a systematic review and meta-analysis". American Journal of Respiratory and Critical Care Medicine 203, Nr. 9 (2021). https://doi.org/10.1164/ajrccm-conference.2021.203.1_MeetingAbstracts.A2578.	Liver
21	Simonetti R G, Perricone G, Nikolova D, Bjelakovic G, and Gluud C. „Plasma expanders for people with cirrhosis and large ascites treated with abdominal paracentesis". Cochrane Database of Systematic Reviews 2019, Nr. 6 (2019). https://doi.org/10.1002/14651858.CD004039.pub2.	Liver
22	Simonetti R G, Perricone G, and Gluud C. „Albumin for people with liver cirrhosis and bacterial infections". Cochrane Database of Systematic Reviews 2021, Nr. 12 (2021). https://doi.org/10.1002/14651858.CD014636.	Liver

23	Sridharan K and Sivaramakrishnan G. „Vasoactive Agents for Hepatorenal Syndrome: A Mixed Treatment Comparison Network Meta-Analysis and Trial Sequential Analysis of Randomized Clinical Trials“. Journal of General Internal Medicine 33, Nr. 1 (2018): 97–102. https://doi.org/10.1007/s11606-017-4178-8.	Liver
24	Teh KB, Loo JH, Tam YC, and Wong YJ. „Efficacy and safety of albumin infusion for overt hepatic encephalopathy: A systematic review and meta-analysis.“ Digestive and liver disease : official journal of the Italian Society of Gastroenterology and the Italian Association for the Study of the Liver 53, Nr. 7 (Juli 2021): 817–23. https://doi.org/10.1016/j.dld.2021.04.030.	Liver
25	Thomson MJ, Taylor A, Sharma P, Lok AS, and Tapper EB. „Limited Progress in Hepatorenal Syndrome (HRS) Reversal and Survival 2002-2018: A Systematic Review and Meta-Analysis.“ Digestive diseases and sciences 65, Nr. 5 (Mai 2020): 1539–48. https://doi.org/10.1007/s10620-019-05858-2.	Liver
26	Velez J C.Q, Befeler A, Kurtz I, Gallegos-Orozco J F, Vargas H E, Vierling J M, Pappas S C, and Jamil K. „Terlipressin improves renal replacement therapy-free survival in hepatorenal syndrome type 1“. Journal of the American Society of Nephrology 31 (2020): 15. https://www.embase.com/search/results?subaction=viewrecord&id=L633697763&from=export.	Liver
27	Velez J C.Q, Sclair S, Sanchez A J, Caldwell S, Sigal S, Thuluvath P J, Satoskar R, Pappas S C, and Jamil K. „Treatment of hepatorenal syndrome type 1 with terlipressin reduces need for renal replacement therapy after liver transplantation“. Journal of the American Society of Nephrology 31 (2020): 75. https://www.embase.com/search/results?subaction=viewrecord&id=L633698106&from=export.	Liver
28	Wong Y J, Qiu T Y, Tam Y C, Mohan B P, Gallegos-Orozco J F, and Adler D G. „Efficacy and safety of intravenous albumin for non-spontaneous bacterial peritonitis infection among patients with cirrhosis: A systematic review and metaanalysis of randomized controlled trials“. United European Gastroenterology Journal 8, Nr. 8 SUPPL (2020): 615. https://doi.org/10.1177/2050640620927345.	Liver
29	Xu T, Liu W, and Huang R. „Can albumin reduce the mortality of patients with cirrhosis and ascites? A meta-analysis of randomized controlled trials.“ European journal of gastroenterology & hepatology 35, Nr. 1 (Januar 2023): 80–88. https://doi.org/10.1097/MEG.0000000000002447.	Liver
30	Zaccherini G, Tufoni M, and Bernardi M. „Albumin administration is efficacious in the management of patients with cirrhosis: A systematic review of the literature“. Hepatic Medicine: Evidence and Research 12 (2020): 153–72. https://doi.org/10.2147/HMER.S264231.	Liver
31	Zheng YJ, Zhuo SJ, Huang B, and Su S. „A meta-analysis of the efficacy and safety of human serum albumin treatment in patients with ascites due to cirrhosis undergoing drainage.“ Asian journal of surgery 44, Nr. 8 (August 2021): 1116–17. https://doi.org/10.1016/j.asjsur.2021.05.040.	Liver
32	Belousov A. „Fundamental knowledge about the physical and chemical properties of commercial albumin and its application in clinical practice“. International Medicine 1, Nr. 3 (2020): 116–21. https://doi.org/10.5455/im.48352.	No Meta-analysis
33	Frederick R T, Lim N, Khan M, Zafar Z, and Jamil K. „Treatment Response to Terlipressin Plus Albumin Varies by Precipitating Factor in Patients With	No Meta-analysis

	Hepatorenal Syndrome Type 1“. American Journal of Gastroenterology 117, Nr. 10 (2022): S865–66. https://doi.org/10.14309/01.ajg.0000861404.38614.53.	
34	Frederick R T, Pappas C, and Jamil K. „Gender affects the association between serum creatinine levels and clinical response to terlipressin in patients with hepatorenal syndrome type of acute kidney injury“. Journal of Hepatology 77 (2022): S611. https://doi.org/10.1016/S0168-8278(22)01538-0.	No Meta-analysis
35	Haynes G R and Bassiri K. „Hyper-oncotic vs. Hypo-oncotic Albumin Solutions: a Systematic Review of Clinical Efficacy and Safety“. SN Comprehensive Clinical Medicine 3, Nr. 5 (2021): 1137–47. https://doi.org/10.1007/s42399-021-00755-0.	No Meta-analysis
36	Kulkarni A V, Da P K, Sharma M, Talukdar R, Reddy N D, and Rao P N. „Midodrine is superior to albumin in prevention of paracentesis induced circulatory dysfunction-a systemic review and network meta-analysis“. Hepatology v70 suppl.1 2019 70 (2019): 197A-198A. https://www.embase.com/search/results?subaction=viewrecord&id=L631811588&from=export.	No Meta-analysis
37	Matera M G, Rogliani P, Bianco A, and Cazzola M. „Pharmacological management of adult patients with acute respiratory distress syndrome“. Expert Opinion on Pharmacotherapy 21, Nr. 17 (2020): 2169–83. https://doi.org/10.1080/14656566.2020.1801636.	No Meta-analysis
38	Wagener G, Bezinover D, Wang C, Kroepfl E, Diaz G, Giordano C, West J, et al. „Fluid Management during Kidney Transplantation: A Consensus Statement of the Committee on Transplant Anesthesia of the American Society of Anesthesiologists“. Transplantation, 2021, 1677–84. https://doi.org/10.1097/TP.0000000000003581.	No Meta-analysis
39	Ho J J, Adnan A S, Kueh Y C, Ambak N J, Van Rostenberghe H, and Jummaat F. „Human albumin infusion for treating oedema in people with nephrotic syndrome“. Cochrane Database of Systematic Reviews 2019, Nr. 7 (2019). https://doi.org/10.1002/14651858.CD009692.pub2.	No Meta-analysis
40	Wiedermann CJ. „Human albumin and 6% hydroxyethyl starches (130/0.4) in cardiac surgery: a meta-analysis revisited.“ BMC surgery 22, Nr. 1 (April 2022): 140. https://doi.org/10.1186/s12893-022-01588-x.	No Meta-analysis
41	Li B, Zhao H, Zhang J, Yan Q, Li T, and Liu L. „Resuscitation Fluids in Septic Shock: A Network Meta-Analysis of Randomized Controlled Trials“. Shock 53, Nr. 6 (2020): 679–85. https://doi.org/10.1097/SHK.0000000000001468.	Network / umbrella meta-analysis
42	Tseng C H. „FLUID RESUSCITATION IN SEPSIS, TRAUMA, TRAUMATIC BRAIN INJURY AND BURN INJURY PATIENTS: NETWORK META-ANALYSIS“. Chest 155, Nr. 4 (2019): 117A. https://doi.org/10.1016/j.chest.2019.02.116.	Network / umbrella meta-analysis
43	Wang L, Long Y, Li K X, and Xu G S. „Pharmacological treatment of hepatorenal syndrome: A network meta-analysis“. Gastroenterology Report 8, Nr. 2 (2020): 111–18. https://doi.org/10.1093/gastro/goz043.	Network / umbrella meta-analysis
44	Zheng X, Bai Z, Wang T, Romeiro F G, Mancuso A, Philips C A, Wong Y J, Nery F G, and Qi X. „Human Albumin Infusion for the Management of Liver Cirrhosis and Its Complications: An Overview of Major Findings from Meta-analyses“. Advances in Therapy 40, Nr. 4 (2023): 1494–1529. https://doi.org/10.1007/s12325-023-02430-3.	Network / umbrella meta-analysis
45	Yang B, Liu S, Qian Z, and Tong Z. „Predicting the death of patients with anti-melanoma differentiation-associated protein-5-positive dermatomyositis-	No target population

	associated interstitial lung disease: A systematic review and meta-analysis“. Modern rheumatology, 2023. https://doi.org/10.1093/mr/road042.	
46	Pan F, Wang Y X, Xie J, Liu Y X, and He X. „Effect of Pre-exchange Transfusion Albumin Infusion on Neonatal Hyperbilirubinemia: A Meta-analysis of Randomized Clinical Trials“. Iranian Journal of Pediatrics 32, Nr. 6 (2022). https://doi.org/10.5812/ijp-129271.	No target population
47	Kao Y, Loh E W, Hsu C C, Lin H J, Huang C C, Chou Y Y, Lien C C, and Tam K W. „Fluid Resuscitation in Patients With Severe Burns: A Meta-analysis of Randomized Controlled Trials“. Academic Emergency Medicine 25, Nr. 3 (2018): 320–29. https://doi.org/10.1111/acem.13333.	Albumin not given
48	Messina A, Robba C, Calabrò L, Zambelli D, Iannuzzi F, Molinari E, Scarano S, et al. „Association between perioperative fluid administration and postoperative outcomes: a 20-year systematic review and a meta-analysis of randomized goal-directed trials in major visceral/noncardiac surgery“. Critical Care 25, Nr. 1 (2021). https://doi.org/10.1186/s13054-021-03464-1.	Albumin not given
49	Tseng CH, Chen TT, Wu MY, Chan MC, Shih MC, and Tu YK. „Resuscitation fluid types in sepsis, surgical, and trauma patients: a systematic review and sequential network meta-analyses.“ Critical care (London, England) 24, Nr. 1 (Dezember 2020): 693. https://doi.org/10.1186/s13054-020-03419-y.	Double report
50	Wong YJ, Qiu TY, Tam YC, Mohan BP, Gallegos-Orozco JF, and Adler DG. „Efficacy and Safety of IV albumin for non-spontaneous bacterial peritonitis infection among patients with cirrhosis: A systematic review and meta-analysis.“ Digestive and liver disease : official journal of the Italian Society of Gastroenterology and the Italian Association for the Study of the Liver 52, Nr. 10 (Oktober 2020): 1137–42. https://doi.org/10.1016/j.dld.2020.05.047.	Double report

Table S2. Methodological quality of the included meta-analyses by AMSTAR-2

	Question	**Cha-marthi et al.** [20]	**Geng et al.** [13]	**Huang & Xiao** [16]	**Itagaki et al.** [17]	**Kesh-avarz et al.** [19]	**Lee et al.** [15]	**Lewis et al.** [21]	**Martin & Bassett** [18]	**Siemens et al.** [22]	**Wei et al.** [14]	**Wiedermann** [23]	**Zou et al.** [23]
1	Did the research questions and inclusion criteria for the review include the components of PICO?	Yes	Yes	Yes	Yes	Yes	Yes	Yes	Yes	Yes	Yes	Yes	Yes
2*	Did the report of the review contain an explicit statement that the review methods were established prior to the conduct of the review and did the report justify any significant deviations from the protocol?	No	No	No	Yes	No	Yes	Yes	No	No	No	No	No
3	Did the review authors explain their selection of the study designs for inclusion in the review?	Yes	Yes	Yes	Yes	Yes	Yes	Yes	Yes	Yes	Yes	Yes	Yes
4*	Did the review authors use a comprehensive literature search strategy?	n.d.	Partial yes	No	Yes	No	Yes	Yes	Partial yes	Yes	Partial Yes	Partial yes	Yes
5	Did the review authors perform study selection in duplicate?	n.d.	Yes	Yes	Yes	n.d.	Yes	Yes	Yes	Yes	Yes	No	Yes
6	Did the review authors perform data extraction in duplicate?	n.d.	Yes	Yes	Yes	n.d.	Yes	Yes	No	Yes	Yes	No	Yes
7*	Did the review authors provide a list of excluded studies and justify the exclusions?	No	No	No	No	No	Yes	Yes	No	No	No	No	Yes
8	Did the review authors describe the included studies in adequate detail?	No	Partial yes	No	Partial yes	No	Partial yes	Yes	Partial yes	Partial Yes	Partial Yes	Partial yes	Partial yes
9*	Did the review authors use a satisfactory technique for assessing the risk of bias (RoB) in individual studies that were included in the review?	No	Partial yes	Partial yes	Yes	n.d.	Yes	Yes	Yes	Yes	Yes	No	Yes
10	Did the review authors report on the sources of funding for the studies included in the review?	No	No	No	No	No	No	Yes	No	No	No	No	No
11*	If meta-analysis was performed did the review authors use appropriate methods for statistical combination of results?	n.d	Yes	Yes	Yes	Yes	Yes	Yes	Yes	Yes	Yes	Yes	Yes
12	If meta-analysis was performed, did the review authors assess the potential impact of RoB in individual studies on the results of the meta-analysis or other evidence synthesis?	n.d.	No	Yes	Yes	n.d.	Yes	Yes	Yes	Yes	Yes	Yes	Yes

13*	Did the review authors account for RoB in individual studies when interpreting/ discussing the results of the review?	No	No	Yes	Yes	No	Yes	Yes	Yes	Yes	Yes	Yes	Yes
14	Did the review authors provide a satisfactory explanation for, and discussion of, any heterogeneity observed in the results of the review?	No	Yes	Yes	Yes	No	Yes	Yes	Yes	No	No	Yes	Yes
15*	If they performed quantitative synthesis did the review authors carry out an adequate investigation of publication bias (small study bias) and discuss its likely impact on the results of the review?	No	No	Yes	Yes	No	Yes	Yes	No	No	Yes	Yes	Yes
16	Did the review authors report any potential sources of conflict of interest, including any funding they received for conducting the review?	No	Yes	Yes	Yes	No	Yes	Yes	Yes	Yes	Yes	Yes	Yes
	Quality	Critically low	Critically low	Critically low	Low	Critically low	High	High	Critically low	Critically low	Critically low	Critically low	Low

* Critical domain. n.d., not documented.

Table S3. Search strategies

DATABASE	SEARCH STRATEGY
PUBMED	("albumin s"[All Fields] OR "albumine"[All Fields] OR "albumines"[All Fields] OR "albumins"[Supplementary Concept] OR "albumins"[All Fields] OR "albumin"[All Fields] OR "albumins"[MeSH Terms]) AND ((y_5[Filter]) AND (meta-analysis[Filter]))
EMBASE	('albumin'/exp OR albumin) AND ((2018:py OR 2019:py OR 2020:py OR 2021:py OR 2022:py OR 2023:py OR 2024:py) AND 'meta analysis'/de)).
COCHRANE DATABASE OF SYSTEMATIC REVIEW	('systematic review' OR 'systematic literature review' OR 'meta-analysis' OR 'meta-analyses' OR 'meta analysis' OR 'meta analyses') AND (albumin) - with Cochrane Library publication date from Jan 2018 to Jun 2023 in Cochrane Reviews
